# An Amperometric Biosensor for Glucose Determination Prepared from Glucose Oxidase Immobilized in Polyaniline-Polyvinylsulfonate Film

**DOI:** 10.3390/s110808152

**Published:** 2011-08-22

**Authors:** Fatma Arslan, Selvin Ustabaş, Halit Arslan

**Affiliations:** 1 Department of Chemistry, Faculty of Sciences, Gazi University, Ankara 06500, Turkey; E-Mail: halit@gazi.edu.tr; 2 Department of Chemistry, Institute of Sciences, Gazi University, Ankara 06500, Turkey; E-Mail: selvin_kmy@hotmail.com

**Keywords:** glucose, biosensor, amperometry, polyaniline, polyvinylsulphonate

## Abstract

In this study, a novel amperometric glucose biosensor with immobilization of glucose oxidase on electrochemically polymerized polyaniline-polyvinylsulphonate (Pani-Pvs) films has been accomplished via the entrapment technique. Electropolymerization of aniline on the Pt surface of the Pt electrode was carried out at constant potential (0.75 V, *vs.* Ag/AgCl) using an electrochemical cell containing aniline and polyvinylsulphonate. Firstly, the optimum working conditions for preparing polyaniline-polyvinylsulfonate films were investigated. Determination of glucose was carried out by the oxidation of enzymatically produced H_2_O_2_ at 0.4 V *vs.* Ag/AgCl. The effects of pH and temperature were investigated and the optimum pH value was found to be 7.5. The storage stability and operational stability of the enzyme electrode were also studied. The results show that 75% of the response current was retained after 16 activity assays. The prepared glucose biosensor retained 80.6% of initial activity after 40 days when stored in 0.1 M phosphate buffer solution at 4 °C.

## 1. Introduction

Determination of glucose is essential due to its clinical and industrial importance. Rapid determination of blood sugar is very important for treatment and control of diabetes. Thus; numerous efforts have been devoted to the development of glucose biosensors with fast and accurate response. The immobilization technique for localizing an enzyme at the surface of various electrodes plays a very important role in research on glucose biosensors. Conducting polymers are being widely used in biosensor applications because they provide stable and porous matrices for biocomponent immobilization and they also facilitate the electron transfer process. The most widely used conducting polymers for immobilization of enzymes are polyaniline, polypyrrole, polythiophene *etc.* [1–5].

Most electrochemical glucose biosensors are based on the glucose oxidase (GOx) enzyme, which catalyzes the oxidation of glucose to gluconolactone which is hydrolyzed to gluconic acid and hydrogen peroxide. The quantification of glucose can be achieved via electrochemical detection of the enzymatic release of H_2_O_2_ [6,7]:

$$\begin{array}{l} \text{Glucose}+O_{2}\to H_{2}O_{2}+\text{gluconic acid}\\ H_{2}O_{2}\to O_{2}+2H^{+}+2e^{-} \end{array}$$

In this study, first the optimum working conditions to prepare the polyaniline-polyvinylsulphonate (Pani-Pvs) film were investigated. Then we report the immobilization of glucose oxidase onto the Pani-PVS film for determination of glucose via an entrapment procedure. Effects of the immobilization process on kinetic parameters, storage and operational stability of the enzyme were investigated. The optimum working conditions of the biosensor with respect to the substrate concentrations, the pH and temperature were investigated.

## 2. Experimental Section

### 2.1. Equipment and Reagents

The electrochemical studies were carried out using an Epsilon EC electrochemical analyzer with a three-electrode cell. The working electrode was a Pt plate (0.5 cm^2^). The auxiliary and the reference electrodes were a Pt wire and Ag/AgCl electrode (3 M KCl), respectively. The pH values of the buffer solutions were measured with an OrionModel 720A pH/ionmeter. Temperature control was achieved with a Grant W14 thermostat. Glucose oxidase (EC 1.1.3.22, purified from *aspergillus niger* and with an activity of 10 unit mL^−1^) and glucose were purchased from Sigma. Aniline and sodium polyvinylsulphonate (Pvs) were supplied by Merck and Aldrich, respectively. All other chemicals were obtained from Sigma. All the solutions were prepared using double distilled water.

### 2.2. The Electropolymerization of Aniline in the Presence of Polyvinylsulphonate

The surface of the Pt was mechanically, chemically, and electrochemically pretreated prior to the coating process. The surface was first polished with silicon carbide 1200/P2500 grinding paper and subjected to a Bunsen flame. It was then dipped in acetonitrile, ethanol, concentrated HCl, and concentrated nitric acid for 5 min each. The surface was then washed thoroughly with double distilled water before being scanned in 5 M H_2_SO_4_ between −2.0 and 2.0 V for electrochemical treatment. It was again washed with double distilled water and then with acetone to remove any traces of water which may have remained on the surface. This process was repeated prior to each coverage [8]. The surface of the pretreated electrode was covered with a conducting polyaniline in polyvinylsulphonate media. A 10 mL solution containing 0.2 M aniline, 2.5 mL (25%) sodium polyvinylsulphonate was prepared. The electrode was immersed into this solution and the system was purged with purified argon for 10 min in order to remove any traces of oxygen. The polymerization was carried out by using constant potential of 0.75 V for 1 h (*vs.* Ag/AgCl electrode (3 M KCl)). The electrode was washed with buffer solution after the coating process.

### 2.3. The Response of Platinum/Polyaniline-polyvinylsulphonate (Pt/Pani-Pvs) Electrode to Hydrogen Peroxide

ThePt/Pani-Pvs electrode was immersed into phosphate buffer (0.1 M) of pH 7.5. The solution contained 0.1 M sodium perchlorate as supporting electrolyte. The electrode was brought to equilibrium by keeping at 0.4 V. Steady current (i_a_) was recorded. Hydrogen peroxide was added to the cell from a stock solution by using a micropipette, and the system was stirred. The currents (i_b_) obtained at 0.4 V were recorded. The current values (Δi = i_b_ − i_a_) were plotted against the hydrogen peroxide concentration.

### 2.4. Preparation of Glucose Biosensor

Immobilization of glucose oxidase was carried out by the physical entrapment technique. Pt/Pani-Pvs electrode was immersed in 0.2 M aniline and 2.5 mL aqueous solution of the sodium salt of polyvinylsulphonate (25%). Then 1 mL of GOX enzyme (10 U/mL) was added into the solution, which was purged with argon for the removal of oxygen before electropolymerization. The polymerization was carried out at constant potential of 0.75 V for 60 min [*vs.* Ag/AgCl electrode (3 M KCl)]. After entrapping the glucose oxidase onto the Pani-Pvs film, the electrode was rinsed with deionized water to remove the unreacted aniline monomer and free glucose oxidase. Immobilized enzyme electrodes were kept in a refrigerator at the 4 °C in phosphate buffer when not in use.

### 2.5. Amperometric Biosensor Measurements

Amperometric response studies carried out in phosphate buffer (0.1 M pH 7.5). Operational stability, storage stability, pH and temperature were determined via application of 0.4 V [*vs.* Ag/AgCl electrode (3 M KCl)] by detecting the H_2_O_2_ oxidation current. After the background current reached a stable value, glucose solution was added to the cell using a micropipette and it was stirred. Then the resulting current difference was recorded. Research into the operational and storage stability as well as effects of pH and temperature were carried out using 1.0 × 10^−5^ M concentrations of glucose.

## 3. Results and Discussion

In this study a new glucose sensitive amperometric enzyme electrode was prepared using polyaniline-polyvinylsulphonate film. The parameters effecting to the performance of the biosensor were investigated.

### 3.1. Obtaining Potential of Polyaniline-Polyvinylsulphonate (Pt/Pani-Pvs) Film

**Figure 1.**
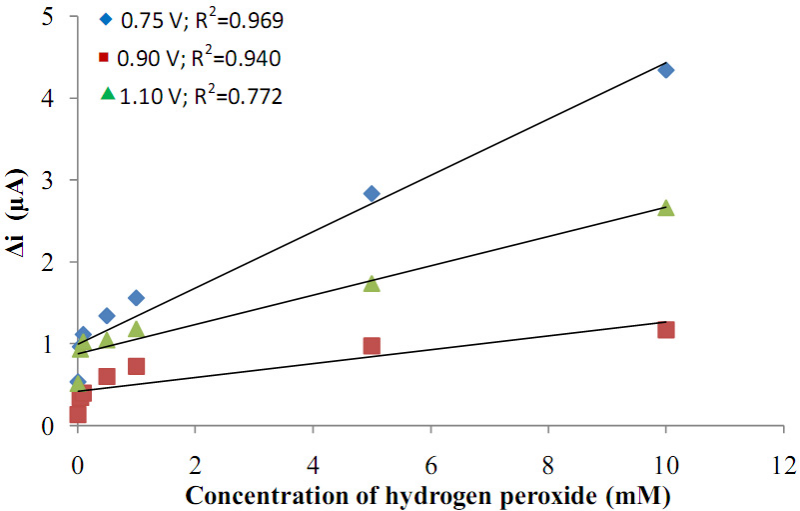
Potential of Pt/Pani-Pvs film *vs*. H_2_O_2_ concentration.

The platinum electrode was covered with polyaniline at different potentials (0.75 V; 0.95 V and 1.10 V) in polyvinylsulphonate media. After the coating procedure, H_2_O_2_ was added to determine the sensitivity of the electrode to H_2_O_2_. A graphic of the response currents of the Pt/Pani-Pvs electrode against H_2_O_2_ concentration was drawn (Figure 1). When the graph was examined, it was seen that current values in 0.75 V were higher than the current values in other potentials and the linearity of the curve which was drawn in 0.75 V was better than the other potentials. The film thickness produced at 1.10 V was more than at other voltages. Because the thickness of the film is so high, conductivity decreased and thus currents became lower. For that reasons, the covering potential was chosen as 0.75 V at which the highest currents and correlation coefficient were obtained.

### 3.2. The Effect of Aniline Concentration

**Figure 2.**
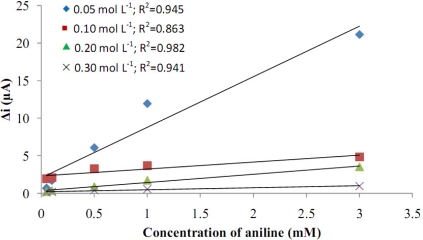
Effect of aniline concentration on the response of the Pt/Pani-Pvs electrode to hydrogen peroxide.

The electrodes’ sensitivity to hydrogen peroxide was investigated in order to elucidate the role of aniline concentration (Figure 2). Four different aniline concentrations (0.05, 0.1, 0.2, 0.3 M) were used. Figure 2 indicates that the current response of the electrode covered in 0.3 M aniline was the lowest, and the surface of this electrode was not homogeneous. The polymer coverage easily peeled from the surface. It was concluded that a high aniline concentration was not suitable for polymer coverage. When the aniline concentration was 0.05 M, it was seen that the current was high. It was observed that the thickness of the polymer formed on the Pt surface by 0.05 M aniline was very thin. During the experiment, as the hydrogen peroxide concentration was increased, the polymer film was becoming thinner and the bare Pt surface came out. The surface deformation could be seen with the naked eye. Because of that, the current values were high in the covers made in 0.05 M concentration. The highest correlation coefficient was observed in 0.2 M pyrrole. For the electrodes prepared by 0.2 M aniline, the surface of the electrode was more homogeneous and stronger than the others. Because of that, the aniline concentration was taken as 0.2 M in the subsequent studies.

### 3.3. The Effect of Thickness of Polyaniline-Polyvinylsulphonate Film

**Figure 3.**
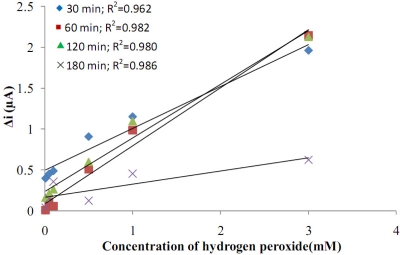
Effect of the film thickness on the response of the Pt/Pani-Pvs electrode to hydrogen peroxide.

The effects of film thickness on the response currents of the electrodes against hydrogen peroxide concentration were investigated (Figure 3). By growing the polymer on the platinum electrode for different time periods (30, 60, 120, 180 min), electrodes with different film thicknesses were prepared. It was observed that the thickness of the polymer formed on the Pt surface in 30 min was too thin. During the experiment, as the hydrogen peroxide concentration was increased, the polymer film was getting thinner and the bare Pt surface was exposed. The correlation coefficient for the 180 min coverage was high, but currents were very low. The thickness of the polymers formed on the Pt surface in 120 and 180 min were too thick and easily broke down and contaminated the solution. Because of this, they could not be used. For the electrodes prepared in 60 min, the surface of the electrode was more smooth and strong than the others. They also had much more mechanical strength and reproducibility. Also, the correlation coefficient of the graphic which was drawn for the film that was covered for 60 min, was highest. Because of these reasons, 60 min covering time was used.

### 3.4. The Determination of Working Potential

**Figure 4.**
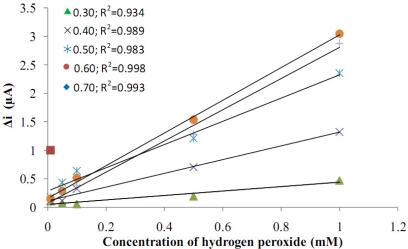
The effect of potential on the response of the Pt/Pani-Pvs electrode to hydrogen peroxide.

After preparing Pt/Pani-Pvs electrodes, the hydrogen peroxide oxidation was carried out at different potentials (0.10, 0.20, 0.30, 0.40, 0.50, 0.60, 0.70 V, Figure 4). Current differences of H_2_O_2_ (0.1 mM) at the different potentials were measured using a Pt/Pani-Pvs electrode and plotted against potential.

When the graphic was examined, it was seen that the variation in current was higher at high potentials than at low potentials. In the studies which were made in 0.10 V and 0.20 V, current values were observed to be nearly zero. Because of this, the current values that were measured at these potentials haven’t been shown in the graphic. It was seen that the linearity of curves at high potentials was higher. It is known that interference effects of the substances present in body fluids (e.g., ascorbic acid, uric acid) are more significant at high potentials [9]. Because of this, even though the linearity of curves at high potentials was higher, these potentials weren’t chosen as working potentials. At 0.30 V, a potential where interference effects could be low, the correlation coefficient was low. Because of the correlation coefficient of the line occurring at 0.40 V was better, 0.40 V were chosen as the working potential.

### 3.5. The Effect of pH on the Response of the Polyaniline-Polyaniline-Polyvinylsulphonate Film to Hydrogen Peroxide

**Figure 5.**
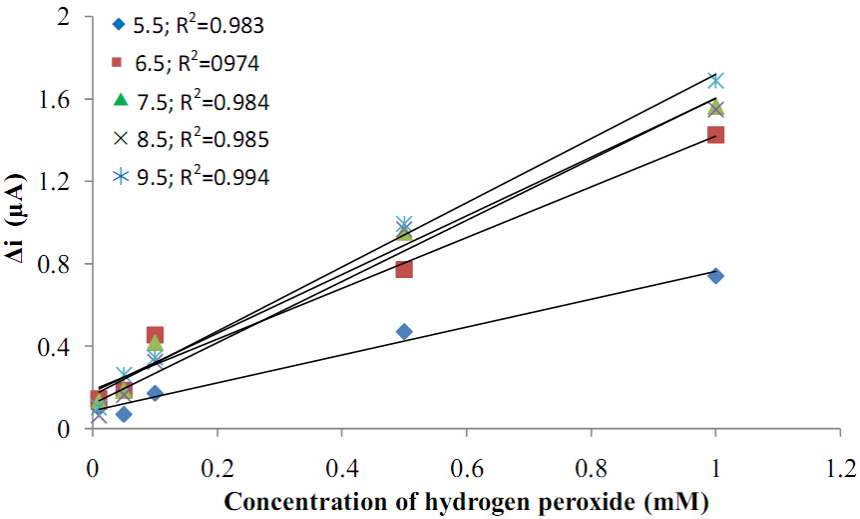
Effect of pH on the response of the Pt/Pani-Pvs electrode to hydrogen peroxide.

The sensitivity of the electrode to H_2_O_2_ was investigated at five different pH values (5.5, 6.5, 7.5, 8.5, 9.5). Current values that were obtained were drawn against H_2_O_2_ concentration. When Figure 5 was examined, it was seen that the H_2_O_2_ anodic currents increased with the increase in pH value. The lowest anodic currents were seen at pH = 5.5. Anodic currents after pH = 6.5 were much higher, but there weren’t any very big changes in currents between the pH values 6.5–9.5. As a result, the pH values after 6.5 could be more suitable for the biosensors made with Pani-Pvs film.

The optimum conditions for preparation of Pt/Pani-Pvs electrode are summarized in Table 1.

### 3.6. The Effect of pH on Amperometric Response of Biosensors

**Figure 6.**
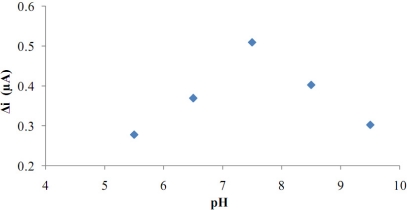
The effect of pH on the response of the biosensor (at 25 °C, 1.0 × 10^−5^ M glucose at 0.4 V operating potential).

Buffer (phosphate buffer) solutions at various pH values were tested to investigate the effect of pH. The pH values of the buffer solutions were varied between 5.5 and 9.5. The measurements were performed at a constant glucose concentration of 1.0 × 10^−5^ M. Figure 6 shows that the maximum response was obtained at pH 7.5. For glucose biosensors pH values other than 7.5 were employed in the literature (pH 6.5; 7.4) [10,11]. This was attributed to the fact that the polymer used and the type of immobilization were different.

### 3.7. The Effect of Temperature

**Figure 7.**
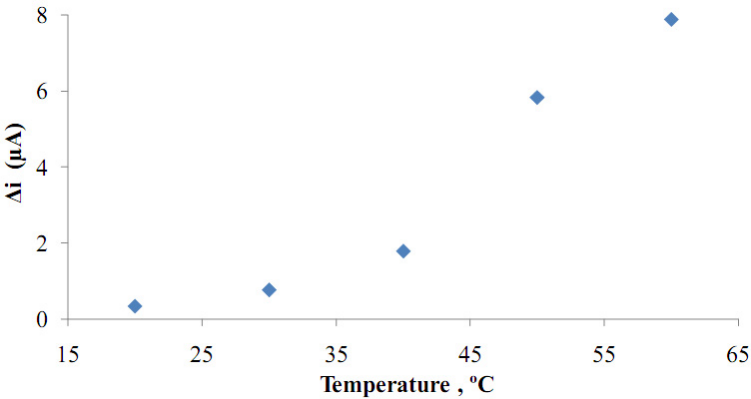
The effect of temperature on the response of the biosensor (at pH 7.5, 1.0 × 10^−5^ M glucose at 0.4 V operating potential).

Temperature has a great effect on enzyme activity and it is important to investigate the temperature’s dependence of the response of the enzyme electrode. The temperature’s influence on the response of glucose enzyme electrode was tested between 20 °C and 60 °C at pH 7.5 using a constant glucose concentration of 1.0 × 10^−5^ M (Figure 7).

When Figure 7 was examined, it was seen that the response currents increased with temperature. Because of the continuous increase in temperature, an optimum value could not be determined. Different temperature values (33 °C and 42 °C) were employed in literature for glucose biosensors [10,12].

When these literatures were examined, it was seen that the polyaniline film, which was obtained by the electropolimerization of aniline, became a good microenvironment around the enzyme. It was observed that the enzyme was stronger, even in high temperatures, because of this microenvironment [13,14]. Therefore, the temperature of 25 °C was chosen as working temperature for all further experiments.

### 3.8. Substrate Concentration and Calibration Curves

**Figure 8.**
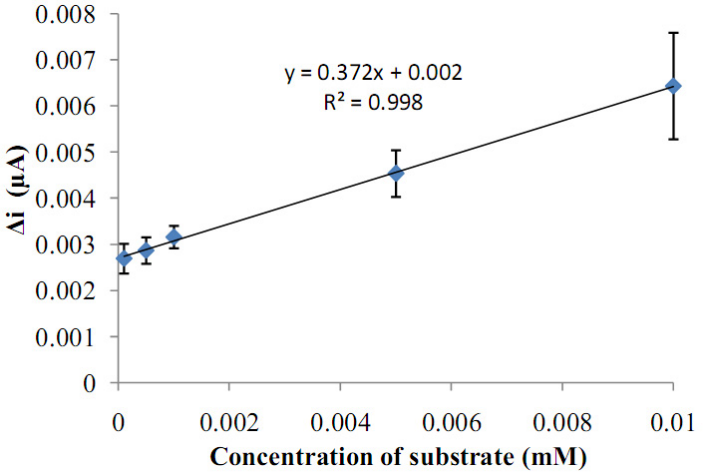
The calibration curve of the glucose biosensor (pH 7.5 phosphate buffer, 25 °C).

**Figure 9.**
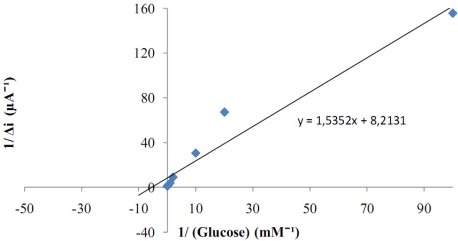
The effect of glucose concentration upon the amperometric response of the biosensor (Lineweaver-Burk plot, in pH 7.5 phosphate buffer and at a 0.4 V operating potential, 25 °C).

There are linear parts ranging between 1.0 × 10^−7^–1.0 × 10^−5^ M (R^2^: 0.999). The graph for the calibration curve was given in Figure 8. It was shown that the linearity of graph was highly satisfactory and it could be used for the quantitative determination of glucose. The detection limit of the biosensor was 1.0 × 10^−7^ M and the response time of the biosensor was 200 s. Kinetic parameters K_m_(app)__ and I_max_ for the enzyme biosensor were detected at constant temperature (25 °C) and pH (pH 7.5) for varying substrate concentrations. K_m_(app)__ and I_max_ were calculated as 0.186 mM, 0.121 μA/min respectively by using 1/[glucose]−1/Δi graph (Lineweaver-Burk plot) (Figure 9). K_m_ values for immobilized glucose oxidase presented in the literature are 18.0, 11.9, 9.34 mM [10,15,16]. This was attributed to the fact that the polymer used and the type of immobilization were different.

### 3.9. Operational Stability and Storage Stability

**Figure 10.**
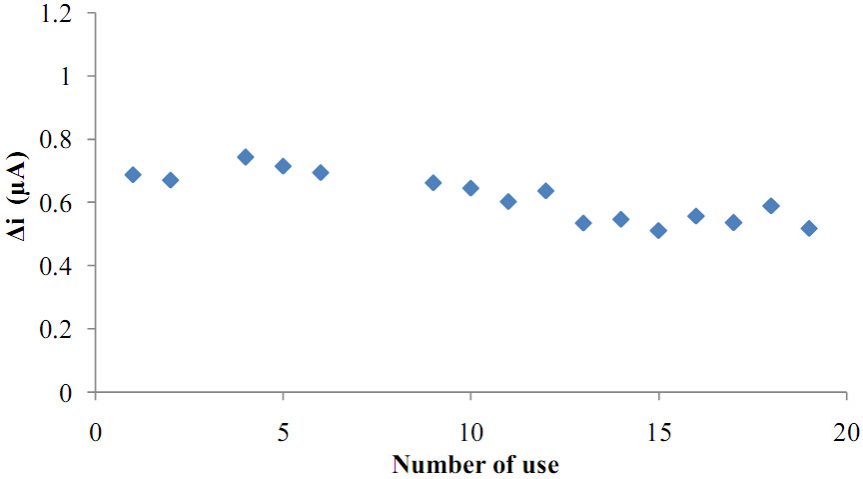
Operational stability of the biosensor in pH 7.5 phosphate buffer, at a 0.4 V operating potential, 25 °C.

The operational stability of the biosensor was studied by performing the activity assay (under optimum conditions) 16 times in the same day at constant temperature, pH and substrate concentration (1.0 × 10^−5^ M). At the end of the 16 measurements, the biosensor had lost 25% of its initial activity (Figure 10). After enzyme immobilization, the enzyme was checked to see if it infiltrated from the pores of the polymer. For this purpose, the enzyme electrode was stored in buffer for a few days. Then, substrate was added to the buffer and the presence of hydrogen peroxide in solution was tested. No hydrogen peroxide signal was observed at 0.4 V. As a result, it was seen that enzyme had not separated from the surface of the electrode. In the entrapment technique, enzyme activity is protected very well. For these reasons, good reproducibility was observed, even for high glucose concentrations.

**Figure 11.**
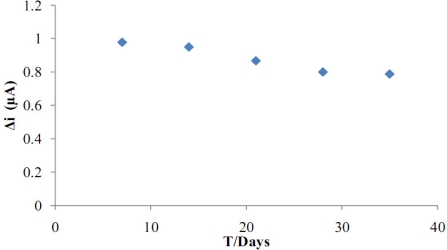
Storage stability of the biosensor (in pH 7.5 phosphate buffer, at 0.4 V operating potential, 25 °C and 1.0 × 10^−5^ M substrate concentration).

Storage stability of the biosensor was determined by performing activity assays within 40 days. The activity assay was applied within 40 days to determine the storage stability of the immobilized enzyme. As shown in Figure 11, an activity loss of 19.4% was observed on the 40th day.

### 3.10. Interference Effect

A few common substances found in serum or urine were studied for any interfering effects on the analysis of glucose. Known concentrations of ascorbic acid, uric acid and paracetamol (acetaminophen) were added. It was observed that a paracetamol concentration of 1.0 × 10^−5^ M had no interfering effects on the analysis of glucose, but the interference effects of ascorbic acid (in 1.0 × 10^−5^ M) and uric acid (in 3.0 × 10^−4^ M) on the analysis of glucose were found to be 15% and 25%, respectively. These interferences were almost removed by dilution of solutions in the cell.

## 4. Conclusions

In this study, glucose oxidase was successfully immobilized on a poly (aniline)-polyvinylsulphonate (Pani-Pvs) composite film. The experimental results clearly showed that the resulting biosensor exhibited good performance in the determination of glucose. It was seen that glucose biosensor was sensitive and its operational stability and long term storage stability were found to be good.

## Figures and Tables

**Table 1.** The optimum conditions for preparation of Pt/Pani-Pvs electrode.

**Parameters**	**Optimum Conditions**
Obtained potential of polyaniline-polyvinylsulphonate film	0.75 V
Aniline concentration	0.2 M
Thickness of polyaniline-polyvinylsulphonate film	Film thickness after covering for 60 min
Working potential	0.4 V
